# Complete mitochondrial genomes and nuclear ribosomal RNA operons of two species of *Diplostomum* (Platyhelminthes: Trematoda): a molecular resource for taxonomy and molecular epidemiology of important fish pathogens

**DOI:** 10.1186/s13071-015-0949-4

**Published:** 2015-06-19

**Authors:** Jan Brabec, Aneta Kostadinova, Tomáš Scholz, D. Timothy J. Littlewood

**Affiliations:** Institute of Parasitology, Biology Centre of the Czech Academy of Sciences and Faculty of Science, University of South Bohemia, Branišovská 31, 370 05 České Budějovice, Czech Republic; Department of Life Sciences, Natural History Museum, Cromwell Road, London, SW7 5BD UK

**Keywords:** *Diplostomum* (Platyhelminthes: Trematoda), Fish pathogens, Mitochondrial genome, Ribosomal RNA, Illumina next-generation sequencing, Phylogeny

## Abstract

**Background:**

The genus *Diplostomum* (Platyhelminthes: Trematoda: Diplostomidae) is a diverse group of freshwater parasites with complex life-cycles and global distribution. The larval stages are important pathogens causing eye fluke disease implicated in substantial impacts on natural fish populations and losses in aquaculture. However, the problematic species delimitation and difficulties in the identification of larval stages hamper the assessment of the distributional and host ranges of *Diplostomum* spp. and their transmission ecology.

**Methods:**

Total genomic DNA was isolated from adult worms and shotgun sequenced using Illumina MiSeq technology. Mitochondrial (mt) genomes and nuclear ribosomal RNA (rRNA) operons were assembled using established bioinformatic tools and fully annotated. Mt protein-coding genes and nuclear rRNA genes were subjected to phylogenetic analysis by maximum likelihood and the resulting topologies compared.

**Results:**

We characterised novel complete mt genomes and nuclear rRNA operons of two closely related species, *Diplostomum spathaceum* and *D. pseudospathaceum*. Comparative mt genome assessment revealed that the *cox1* gene and its ‘barcode’ region used for molecular identification are the most conserved regions; instead, *nad4* and *nad5* genes were identified as most promising molecular diagnostic markers. Using the novel data, we provide the first genome wide estimation of the phylogenetic relationships of the order Diplostomida, one of the two fundamental lineages of the Digenea. Analyses of the mitogenomic data invariably recovered the Diplostomidae as a sister lineage of the order Plagiorchiida rather than as a basal lineage of the Diplostomida as inferred in rDNA phylogenies; this was concordant with the mt gene order of *Diplostomum* spp. exhibiting closer match to the conserved gene order of the Plagiorchiida.

**Conclusions:**

Complete sequences of the mt genome and rRNA operon of two species of *Diplostomum* provide a valuable resource for novel genetic markers for species delineation and large-scale molecular epidemiology and disease ecology studies based on the most accessible life-cycle stages of eye flukes.

**Electronic supplementary material:**

The online version of this article (doi:10.1186/s13071-015-0949-4) contains supplementary material, which is available to authorized users.

## Background

Trematodes trematodes of the genus *Diplostomum* von Nordmann, 1832, represent a diverse group of parasitic flatworms (Neodermata: Platyhelminthes: Trematoda) that has attracted the attention of parasitologists and evolutionary ecologists for a long time, but has always stood in the shade of medically important taxa, notably the closely related human-infecting schistosomes (blood flukes). Species of *Diplostomum* utilise three different hosts to complete their life-cycles (Additional file 1: Figure S1). First intermediate hosts are lymnaeid snails where the dispersive infective stages (free-swimming cercariae) are asexually produced; these identical genetic clones emerge from the snail hosts in vast quantities in the aquatic environment, actively seek and infect second intermediate hosts, a wide range of freshwater fish. Cercariae migrate to the eyes or brain of fish where they develop into a long-lived infective stage (metacercaria). The life-cycle is completed when infected fishes are consumed by the definitive hosts (fish-eating birds) where adults develop and sexual reproduction occurs; eggs are shed, hatch and develop into short-lived free-swimming stages (miracidia), which infect the first intermediate hosts [1].

Due to their wide distributional and host ranges, species of *Diplostomum* represent attractive model systems for population genetics of monoecious parasites with complex life-cycles alternating asexual and sexual reproduction (e.g. [2, 3]) and for studies of host-parasite coevolution in model vertebrate organisms (e.g. [4]). Furthermore, the metacercariae of *Diplostomum* spp. in fish eyes are important pathogens causing diplostomiasis (eyefluke disease of fishes) manifested as various degrees of blindness that affects fish feeding, growth and survival, implicated in substantial impacts on natural populations and losses in aquaculture; this has led to intensive field and experimental studies [1, 5]. However, the model systems used in these studies have been referred to as “*Diplostomum spathaceum*” a collective name for the species found in the lens [5], innominate species of *Diplostomum* [6] or to a composite group of *Diplostomum* spp. [7]. The lack of accurate species identification represents a major impediment in the assessment of transmission dynamics, infectivity and virulence that varies among species and strains of *Diplostomum* [1, 5] and of the effects of these parasites in natural and aquacultured fish populations, as well as in addressing broader questions related to geographical distribution and host ranges of *Diplostomum* spp.

The taxonomy of the genus *Diplostomum* is still in a controversial state due to the lack of unequivocal morphological criteria for species discrimination (see [8] for details) and this has resulted in substantially underestimated species richness in large-scale inventories of natural snail, fish and bird populations. Identification of the larval stages of *Diplostomum* spp. is particularly difficult since linking life-cycle stages requires experimental completion of the life-cycle. The application of DNA-based approaches provides a promising independent method for assessment of species boundaries within the genus and for molecular identification of developmental stages in these parasites with complex life-cycles. The pioneer studies have focused on sequencing of the internal transcribed spacers (ITS1 and ITS1-5.8S-ITS2) of the ribosomal RNA (rRNA) gene cluster [9–11]. However, recent studies have shown that these regions do not provide sufficient resolution for species discrimination within *Diplostomum* and have provided evidence that the barcode region of the mitochondrial (mt) cytochrome *c* oxidase subunit 1 (*cox1*) gene may serve as a more efficient marker in elucidating life-cycles and recognition of cryptic species diversity within *Diplostomum*. Using the diplostomid-specific primers flanking the *cox1* ‘barcode’ region developed by [12], the first molecular prospecting studies predominantly focused on metacercariae in natural fish populations have revealed much higher species diversity than previously estimated from morphology alone in both the Palaearctic and Nearctic [8, 13, 14]. However, the recent expansion of the barcode library for *Diplostomum* spp. revealed low divergence levels within complexes of cryptic species thus hampering unequivocal species identification using the ‘barcode’ *cox1* region alone [8, 14].

To overcome the current limitations, there is a need to supplement the molecular markers available. Characterisation of complete mt genomes represents such an approach whose applicability has already been demonstrated within flatworms of biomedical and veterinary importance (e.g. [15–17]). To greatly alleviate the task of *de novo* characterisation of mt genomes, recent developments in next generation sequencing and downstream bioinformatics have provided a time- and cost-effective strategy to characterise rich amounts of data, out of which mt sequences can be readily identified and mt genomes reconstructed [18]. Here, we demonstrate and discuss the yields of such a strategy, using representatives of two widely distributed species of *Diplostomum* as an example of flatworm parasites of increasing relevance. We characterise the first complete mt genomes and nuclear rRNA operons of two closely related species of the basal digenean order Diplostomida, *Diplostomum spathaceum* and *D. pseudospathaceum*, assess the existing mt markers and identify new regions of the mt genomes that are promising for simultaneous species delineation and large-scale molecular epidemiology assessments. As a by-product of our mt genome sequencing effort, we have characterised the complete transcribed region of the nuclear rRNA operon and provide the first genome wide assessment of the phylogenetic relationships of the order Diplostomida, one of the two fundamental lineages of the platyhelminth subclass Digenea.

## Methods

### DNA sources, sequencing and assembly

Adults of *Diplostomum spathaceum* and *D. pseudospathaceum* were sampled from the intestine of two freshly collected black-headed gulls (*Larus ridibundus*) from Chropyně and Tovačov (Czech Republic). Individual specimens of *Diplostomum* spp. were identified on the basis of their morphology [8], stored separately in absolute ethanol before total genomic DNA was extracted from single adult individuals per species with the use of QIAamp DNA Mini kit (Qiagen, Hilden, Germany) according to the manufacturer’s instructions with the final DNA elution done in two steps, each time using 50 μl of AE buffer (Qiagen). The two eluates were combined and the amount of total DNA isolated was measured with Qubit® 2.0 Fluorometer (Life Technologies, Paisley, UK) yielding 2.1 and 1.3 ng/μl total DNA for *D. spathaceum* and *D. pseudospathaceum*, respectively. Two samples for next generation sequencing (NGS) were prepared and run at the DNA Sequencing Facility of the Natural History Museum (NHM), London, UK. Genomic DNA was indexed and libraries prepared using TruSeq Nano DNA Sample Preparation Kit (Illumina, Inc., San Diego, USA), and run simultaneously with 2 other samples on a MiSeq Illumina sequencer yielding 250 bp long paired-end reads. Three additional individuals of *D. pseudospathaceum* were partially characterised through PCR amplification and Sanger sequencing.

The new mt genomes were directly assembled using the mt baiting and iterative mapping (MITObim) approach of [18]. Partial *cox1* sequences of [8] for *D. spathaceum* (JX986887) and *D. pseudospathaceum* (JX986905) were used as an initial bait to extract the corresponding *cox1* reads from the entire Illumina genomic readpool from which a new reference assembly was subsequently derived. The new reference sequence was then automatically subjected to an iterative set of baiting and mapping steps until the total number of mapped reads became stationary. Resulting MITObim assemblies were then imported in Geneious version 7 [19] where the raw paired-end reads were mapped onto them in a single step using custom sensitivity settings to estimate the full mt genome coverage (do not trim; min. overlap = 25; max. mismatches/read = 4 %). Geneious was then used to trim the overlapping regions to create a circular mt molecule and to inspect for any potential mapping/assembly errors in problematic regions (e.g. the repetitive regions). Two such regions (where the two newly characterised mt genomes markedly differ) were detected and the local assembly fit was manually checked in Geneious through a stepwise iterative mapping of Illumina reads extending from conserved flanking regions.

Contiguous sequences of the nuclear rRNA operon were assembled solely within Geneious through iterative mapping of paired-end reads on the previously published ITS1-5.8S-ITS2 sequences of *D. spathaceum* (JX986844) and *D. pseudospathaceum* (JX986854) from Tovačov (Czech Republic) [8]. Mapping was done through multiple steps using custom sensitivity settings (do not trim; min. overlap = 50; max. mismatches/read = 10 %) and checking the fit of mapped reads by eye.

### Sequence annotation and phylogenetic analyses

**Fig. 1:**
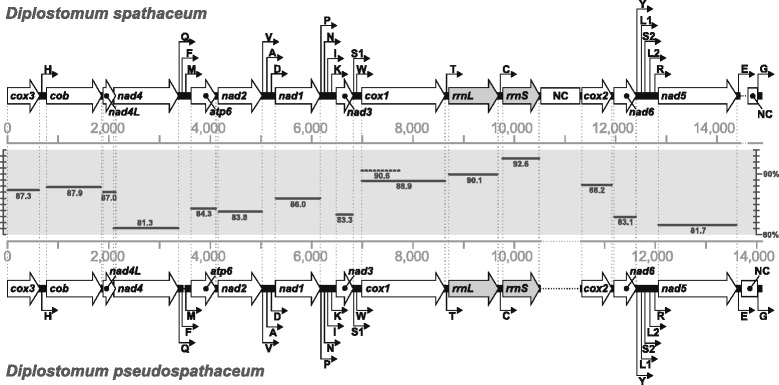
Schematic alignment of linearised mt genomes of *Diplostomum spathaceum* and *D. pseudospathaceum. Outline arrows* indicate the direction of transcription of protein-coding and rRNA genes, *hairline arrows* indicate the position of tRNA genes (see Additional file 4: Table S1 for the single letter abbreviations). *Solid horizontal lines* in the grey central area depict average nucleotide identities of individual protein- and rRNA-coding regions (the ‘barcode’ region of *cox1* defined by Folmer primers indicated by a *dotted line*); *NC* non-coding region

Following the assembly, identity and position of individual mt protein- and tRNA-coding regions were determined using a suite of bioinformatics tools. MITOS [20] was used to reveal the position of mt protein-coding and rRNA genes, whereas tRNAscan-SE web server [21] together with ARWEN [22] were employed to localise tRNA genes and reconstruct their secondary structures. Exact boundaries of the mt protein-coding genes were then confirmed by alignment of inferred amino acid sequences with those of mt genomes of *Trichobilharzia regenti* (NC_009680), *Fasciola hepatica* (NC_002546) and *Schistosoma japonicum* (NC_002544), using E-INS-i aligning algorithm of MAFFT [23] implemented in Geneious. Identical aligning strategy was also used to create pairwise alignment of the whole-length of mt genomes of *D. spathaceum* and *D. pseudospathaceum* (Fig. 1).

Exact coding positions of individual nuclear rRNA genes (rDNA), as well as positions of the affiliated transcribed spacers, were determined progressively through the following series of steps: the entire assemblies were BLAST-searched against sequences of *Schistosoma japonicum* and *Trichobilharzia regenti* in GenBank and coding rDNA and ITS regions aligned with E-INS-i algorithm of MAFFT; exact boundaries of the ITS1 and ITS2 were identified with the program ITSx 1.0.10 [24]; putative endpoints of lsrDNA and 3′ external transcribed spacer (ETS), as well as the rRNA operon transcription start matching the 5′ end of 5′ ETS, were localised according to [25] and [26]; and finally, the complete annotation was compared with the fully-annotated complete human rRNA repeating unit (Accession No. HSU13369; [27]).

**Fig. 2:**
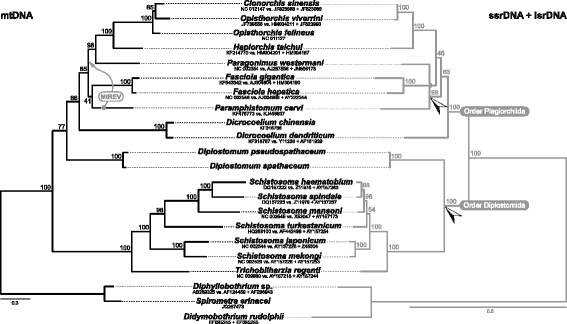
Phylogenetic position of the genus *Diplostomum* estimated under the maximum likelihood criterion based on analyses of mt protein-coding amino acid data (*left*) and nuclear rRNA genes (*right*). Node labels depict bootstrap support calculated from 400 repetitions in RAxML. Rooted phylogram; outgroup: *Diphyllobothrium* sp., *Spirometra erinacei* and *Didymobothrium rudolphii*. Please note the alternative position of *Paramphistomum cervi* based on the mt amino acid data analysis under the MtREV substitution matrix. *Arrows* on the rDNA data-based phylogram indicate nodes in conflict with the mt data-based topology

Phylogenetic position of *Diplostomum* spp. was estimated on the basis of concatenated amino acid and nucleotide sequence data for the two novel and 17 available mt genomes of representatives of both basal (order Diplostomida; seven species) and derived (order Plagiorchiida; ten species) clades of the platyhelminth subclass Digenea, and from an analogous dataset of rDNA sequences (ssrDNA and lsrDNA) made as complete as possible. These included *Schistosoma haematobium*, *S. japonicum, S. mansoni*, S. *mekongi*, *S. spindale*, *S. turkestanicum* and *Trichobilharzia regenti*, all representatives of a single family (Schistosomatidae) of the order Diplostomida, and representatives of six families of the order Plagiorchiida: *Clonorchis sinensis*, *Opisthorchis felineus* and *O. viverrini* (Opisthorchiidae); *Dicrocoelium chinensis* and *D. dendriticum* (Dicrocoeliidae); *Fasciola gigantica* and *F. hepatica* (Fasciolidae); *Haplorchis taichui* (Heterophyidae); *Paragonimus westermani* (Paragonimidae); and *Paramphistomum cervi* (Paramphistomidae). Sequences representing three species of the platyhelminth class Cestoda (*Didymobothrium rudolphii*, *Diphyllobothrium* sp. and *Spirometra erinacei*; GenBank accession numbers for all taxa provided in Fig. 2) were also included. Since entire mt genomes could not be aligned, all 12 protein-coding gene regions were extracted, nucleotide sequences for individual genes each translated into amino acids using the echinoderm and flatworm mt translation code (NCBI Table 9, [28]), aligned with L-INS-i algorithm of MAFFT, manually curated to exclude ambiguously aligned regions, concatenated, and analysed under the MtZoa and MtREV substitution models [29, 30]. The rDNA dataset was aligned with E-INS-i algorithm of MAFFT, manually curated to exclude ambiguously aligned regions, ssrDNA and lsrDNA partitions concatenated and analysed as a single partition under the GTR + I + Γ substitution model. Phylogenetic analyses of the two genomic loci (conducted at both amino acid and nucleotide levels in the case of the mt data) were carried out individually, under the maximum likelihood (ML) criteria in the program RAxML version 8.1.7 [31]. All model parameters and bootstrap nodal support values were estimated using RAxML; the number of bootstrap repetitions was estimated with the extended majority rule (MRE) bootstopping method [32].

## Results

### Characteristics of the mt genomes

Genomic DNA sequencing of *D. spathaceum* and *D. pseudospathaceum* using 1/4 of a MiSeq run each yielded 3.43 and 4.73 million indexed pair-end 250 bp reads, respectively. Of these, two complete mt genomes with overlapping flanking regions were assembled and trimmed to form a consensual circular sequence. The length of the recovered mt genomes of *D. spathaceum* and *D. pseudospathaceum* (GenBank accessions KR269763 and KR269764) was 14,784 and 14,099 bp, respectively. The total number of reads mapped on the trimmed mt genomes was 7762 and 5326, accounting for 0.23 and 0.11 % of the genomic readpool obtained, with minimum coverage of 61 and 53 reads, respectively (see Table 1 for further details).

**Table 1:** Details on the mt genome assemblies of the two species of *Diplostomum* out of Illumina MiSeq shotgun genome sequencing

	MiSeq output (reads per 1/4 run)	mt genome length (bp)	mtDNA nucleotide frequencies (%)				Mapped reads	mtDNA coverage		
			A	C	G	T		min	max	mean
*D. spathaceum*	3,432,950	14,784	22.3	10.6	20.1	47.1	7,762	61	170	131
*D. pseudospathaceum*	4,729,330	14,099	22.4	9.9	19.8	47.9	5,326	53	135	94

The mt genomes of *D. spathaceum* and *D. pseudospathaceum* were fully annotated (Fig. 1), both featuring an identical, complete suite of 12 intron-less protein-coding genes typical for the Platyhelminthes (all lacking the *atp8* gene): ATP synthase subunit 6 (*atp6*), cytochrome *b* (*cob*), three subunits of cytochrome *c* oxidase (*cox1–3*), and seven subunits of NADH dehydrogenase (*nad1–6*, *nad4L*). Further, the two assemblies contained small and large subunits of the mt rRNA (*rrnS*, *rrnL*), and a total of 22 transfer RNA genes (including two tRNA copies for amino acids leucine and serine; see inferred secondary structures in Additional file 2: Figure S2 and Additional file 3: Figure S3). All of these genes were encoded unidirectionally, on the plus strand (Fig. 1), in widespread agreement with the protein- and rRNA-coding backbone gene order observed in all other parasitic flatworms, with the exception of the derived African schistosomes ([17]; see positions and sequence lengths of individual genes in Table 2); *nad4L* overlapped the first 40 nucleotides of *nad4* (Table 2). We found no sign of protein-coding gene duplications. The observed adenine + thymine (AT) content of the mt genomes was high, accounting for 69.3 % in *D. spathaceum* and 70.4 % in *D. pseudospathaceum*. The most AT-rich protein-coding genes were *nad3* (73.7 %) in *D. spathaceum* and *nad4L* (75.0 %) in *D. pseudospathaceum*. In contrast, the lowest AT content was found in *cox2* (67.2 and 64.9 %, respectively).

**Table 2:** Summary data on mt genome organisation of the two species of *Diplostomum*, positions and sequence lengths of individual genes, initiation and termination codons, anticodons and the lengths of predicted proteins

Gene	*D. spathaceum*					*D. pseudospathaceum*				
	Coding position		Length^a^	Start/stop	Anticodon	Coding position		Length^a^	Start/stop	Anticodon
*cox3*	1	655	655	ATG/T		1	655	655	ATG/T	
*trnH*	680	747	68		GTG	680	747	68		GTG
*cob*	751	1861	1111	ATG/T		751	1861	1111	ATG/T	
*nad4L*	1863	2126	264	ATG/TAG		1863	2126	264	ATG/TAG	
*nad4*	2087	3385	1299	ATG/TAG		2087	3382	1296	ATG/TAG	
*trnQ*	3389	3452	64		TTG	3385	3448	64		TTG^b^
*trnF*	3465	3529	65		GAA	3458	3521	64		GAA
*trnM*	3566	3634	69		CAT	3532	3600	69		CAT
*atp6*	3638	4156	519	ATG/TAA		3604	4122	519	ATG/TAA	
*nad2*	4182	5073	892	GTG/T		4139	5039	901	TTG/T	
*trnV*	5074	5136	63		TAC	5040	5102	63		TAC
*trnA*	5146	5212	67		TGC	5115	5178	64		TGC
*trnD*	5246	5310	65		GTC	5212	5276	65		GTC
*nad1*	5312	6221	910	GTG/T		5277	6186	910	GTG/T	
*trnP*	6222	6286	65		TGG	6187	6251	65		TGG
*trnN*	6290	6356	67		GTT	6255	6320	66		GTT
*trnI*	6363	6428	66		GAT^b^	6325	6390	66		GAT
*trnK*	6430	6498	69		CTT	6393	6460	68		CTT
*nad3*	6502	6858	357	GTG/TAG		6465	6821	357	GTG/TAA	
*trnS*	6862	6921	60		GCT^b^	6824	6883	60		GCT^b^
*trnW*	6931	6997	67		TCA	6893	6960	68		TCA
*cox1*	7007	8665	1659	ATG/TAG		6970	8628	1659	ATG/TAA	
*trnT*	8694	8758	65		TGT	8659	8723	65		TGT
*rrnL*	8759	9750	992			8724	9723	1000		
*trnC*	9751	9820	70		GCA	9724	9794	71		GCA
*rrnS*	9821	10545	725			9795	10519	725		
*cox2*	11355	11969	615	ATG/TAA		10548	11162	615	ATG/TAA	
*nad6*	11984	12442	459	ATG/TAG		11175	11633	459	ATG/TAG	
*trnY*	12457	12520	64		GTA	11648	11711	64		GTA
*trnL*	12525	12594	70		TAG	11720	11787	68		AAG
*trnS*	12595	12661	67		TGA	11788	11854	67		TGA
*trnL*	12681	12747	67		TAA	11877	11943	67		TAA
*trnR*	12784	12850	67		TCG	11979	12046	68		TCG
*nad5*	12851	14437	1587	GTG/TAA		12047	13633	1587	GTG/TAA	
*trnE*	14459	14520	62		TTC^b^	13653	13718	66		TTC
*trnG*	14709	14779	71		TCC	14031	14094	64		TCC

^a^Length of protein-coding genes (including stop codons); ^b^tRNAs found only with ARWEN

Codon usage of individual mt protein-coding genes is provided in Additional file 4: Table S1. Most mt protein-coding genes of both *Diplostomum* spp. had ATG as the translation initiation codon whereas *nad1*, *nad2*, *nad3* and *nad5* genes use alternative codons (mostly GTG; Table 2). Surprisingly, *nad2* of *D. pseudospathaceum* starts with a putative TTG, 9 bp upstream of the position of *nad2* start codon GTG in *D. spathaceum*. It seems relatively unlikely that the codon GCA (which corresponds to the position of GTG in *D. spathaceum*) represents the genuine start codon of *nad2* in *D. pseudospathaceum*. We found no evidence of a sequencing error or a single nucleotide polymorphism in this region based on comparisons with three additional partially sequenced adults of *D. pseudospathaceum*. However, direct support from mRNA sequences is required before concluding the presence of an alternative initiation codon in *Diplostomum* spp. Contrary to the start codons, translation termination codons in the two species did not match entirely and the stop codons TAG, TAA and putative truncated T were all found frequently (Table 2).

### Genetic divergence of mt genomes and nuclear rRNA operons

A schematic pairwise alignment of the mt genomes of the two species of *Diplostomum*, depicting the order of the 36 genes and the non-coding regions, is provided in Fig. 1. The entire gene order matched exactly but the two congeneric species differed notably in the total length of the mt circle. The difference stems mainly from the presence of extensive insertions at two loci: a 781 bp long non-coding insertion between *rrnS* and *cox2* genes in *D. spathaceum* and a 124 bp long insertion in *D. pseudospathaceum* found immediately upstream of the common non-coding region situated between *trnE* and *trnG* genes. The insertion in *D. spathaceum* mt genome comprises two conspicuously imperfect repeats (pairwise sequence identity of 79.8 %) not present in the *D. pseudospathaceum* mt genome, and the insert in *D. pseudospathaceum* represents a partly repeated unit found in the 188 bp long non-coding region common to both species. Although surprising, extensive manual checking of assemblies demonstrated that these inserts are genuine. Analysis of pairwise nucleotide sequence identities of the protein-coding and rDNA regions of the mt genomes revealed a range of sequence conservation from levels as low as 81.3 % in *nad4* and 81.7 % in *nad5* to as high as 88.9 % in the most conserved gene *cox1*. The barcode region of *cox1* was even more conserved with 90.6 % of nucleotides of the 722 bp long fragment [33] being identical (Fig. 1). The *rrnL* and *rrnS* gene nucleotide identities reached 90.1 and 92.6 %, respectively.

The transcribed region of the nuclear rRNA operon, although representing only about half the length of the mt genome, mapped 2.4 and 5.2× more MiSeq reads in *D. spathaceum* and *D. pseudospathaceum*, respectively (Table 3). Using the methodological approach of [25] and [26] to supplement a series of multiple sequence alignments and other rDNA annotation strategies (see Methods above), we identified a putative nuclear rRNA operon transcription start site, followed by a 726 bp sequence of 5′ ETS, 1979 bp of ssrDNA, 607–608 bp of ITS1, 157 bp of 5.8S rDNA, 294–295 bp of ITS2, 4210 bp of lsrDNA, and 17 bp long 3′ ETS, forming together a 7991 and 7993 bp long transcribed rDNA region of *D. spathaceum* and *D. pseudospathaceum*, respectively (see Table 3, GenBank accessions KR269765 and KR269766). The total length of the entire operon then likely extends over 9000 bp; however, given the limited length (250 bp) of the raw MiSeq reads and the observed presence of multiple repeat motifs in the ribosomal intergenic spacer (IGS) region separating individual rRNA operons, we refrained from assembling the entire rDNA tandem repeat unit and limited our comparisons to the transcribed portion of the region.

**Table 3:** Details on the assemblies of the transcribed rRNA operon of the two species of *Diplostomum* out of Illumina MiSeq shotgun genome sequencing

	Coding region localisation (bp)			rDNA nucleotide frequences (%)				Mapped reads	Coverage		
	ssrDNA	5.8S	lsrDNA	A	C	G	T		min	max	mean
*D. spathaceum*	728–2706	3315–3471	3767–7976	22.7	22.4	28.9	26.0	18,791	303	834	563
*D. pseudospathaceum*	728–2706	3314–3470	3765–7974	22.6	22.3	29.0	26.1	27,760	476	1131	833

Pairwise alignment of rRNA operons revealed remarkably high levels of nucleotide conservation with coding regions reaching from 99.8 % (ssrDNA) and 99.9 % (lsrDNA) to 100 % (5.8S rDNA) of identical nucleotides and high nucleotide identities for the spacers (97.1 % in 5′ ETS, 98.8 % in ITS1 and ITS2, and 100 % in 3′ ETS). Mapping of the raw pair-end MiSeq reads on the rRNA operon under relaxed settings did not reveal any polymorphic sites within the rDNA except for position 3597 in ITS2 of *D. spathaceum*.

### Phylogenetic analyses

Phylogenetic estimates of species of the Digenea based on mitochondrial and rDNA data for 19 species revealed contradictory topologies concerning, most importantly, the phylogenetic placement of the genus *Diplostomum* (Fig. 2). The analysis based on mt protein-coding genes supported its position as a sister lineage to the order Plagiorchiida (and thus not part of the order Diplostomida), whereas the analysis of nearly complete sequences of rDNA recovered it as a basal lineage of the Diplostomida supporting this branching pattern with maximum bootstrap values. Considering the analyses of mt data, the only topological difference between estimates employing MtZoa and MtREV substitution matrices was the relatively more derived position of *Paramphistomum cervi* under the MtREV model. Analysis of the same mt data at the level of nucleotides resolved the same placement of *Diplostomum* as a sister lineage of the Plagiorchiida and yet another alternative position of *Paramphistomum cervi* as the most basal lineage of the Plagiorchiida (results not shown). In fact, the phylogenetic position of *Paramphistomum cervi* represents the only other difference between the mt- and rDNA-based trees receiving significant statistical support (it forms a sister lineage to *Paragonimus westermani* in the latter) and will remain problematic given the low statistical support in either of the analyses.

**Fig. 3:**
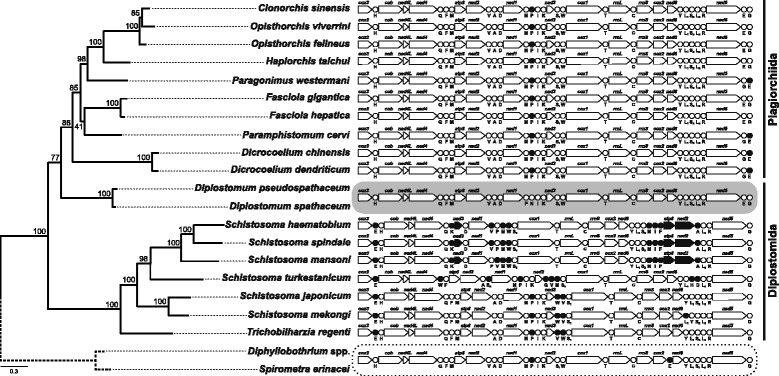
Schematic diagram of the mitochondrial gene order of the Trematoda mapped onto the mtDNA amino acid phylogenetic estimate from Fig. 1. *Diplostomum* spp. gene order highlighted by *grey* background, altered gene position relative to the *Diplostomum* spp. indicated in *black*. Outgroup: *Diphyllobothrium* spp. and *Spirometra erinacei*

Mapping the mt gene arrangement across the phylogenetic tree inferred from mt data revealed that both species of *Diplostomum* exhibit a closer match to the conserved gene order of the plagiorchiid digeneans with only one or two immediately neighbouring tRNAs positions switched (Fig. 3), rather than to the more extensively rearranged order observed in the mt genomes of the representatives of the Diplostomida (basal schistosomes display at least four single tRNA relocations, derived schistosomes a further two shifts of larger genome chunks involving several protein-coding and tRNA genes). The mt gene order in *Diplostomum* spp. resembles most closely that of representatives of the flatworm class Cestoda, a sister lineage of the class Trematoda (e.g. [34]) used as an outgroup in our analyses (Fig. 3).

## Discussion

To the best of our knowledge this is the first application of the NGS approach to completely characterise mt genomes and nuclear rRNA operons of a trematode in a single step. Given the fact that within parasitic flatworms (Neodermata) there have been just two monogenean species of *Gyrodactylus* sequenced using NGS platform (Illumina HiSeq 2000^TM^) to develop an automated *in silico* mt genome assembly approach [18], this is the first use of a NGS platform with limited output to easily characterise multiple-copy genomic loci within parasitic flatworms. We combined a total of four indexed TruSeq Nano library samples (only two samples published here) prepared from untreated DNA extractions on a single Illumina MiSeq run to assess the capability of a simple and straightforward approach to reliably characterise the genomic loci of interest. Of the two loci, nuclear rRNA operons received about five- to nine-fold greater coverage than the mt genomes, whose minimal values in turn were 61 and 53 reads in *D. spathaceum* and *D. pseudospathaceum*, respectively. We consider this a sufficiently high coverage that allows for combining two- or three-fold more flatworm specimens on a single MiSeq run using the same library preparation strategy as employed here in order to ensure that characterisation of complete novel mt genomes and nuclear rRNA operons is as cost-effective as possible while receiving sufficient coverage in future studies.

Our study made available reference mt genomes of closely related *Diplostomum* spp. thus providing a rich resource for future approaches to species delimitation that, in turn, will enable the exploration of molecular epidemiology within a large group of widespread fish pathogens. Recent application of *cox1* ‘barcoding’ proved essential in discovering vast previously unrecognised species diversity within the genus [8, 13, 14]. The currently used primers for amplification of the *cox1* ‘barcode’ region of [12] work reasonably well and seem to allow for differentiation and identification of a number of *Diplostomum* spp. However, problematic low divergence has already been demonstrated within three cryptic species complexes, “*D. baeri*”, “*D. huronense*” and “*D. mergi*” (see [8, 14]). We predict that expansion of the exploration of genetic diversity across host populations will inevitably reveal cryptic species thus making species delineation crucial for defining the host ranges and geographical distribution of *Diplostomum* spp. and the successful development of population genetic studies. Our study profited from the availability of DNA from the adult stages of *Diplostomum* spp. and taxonomic expertise. Given that most of the recently molecularly delimited species-level lineages of *Diplostomum* cannot be formally described (awaiting the collection of the adult stages), we see an increasing need to employ the NGS approach presented here to characterise any morphologically determinable *Diplostomum* specimens available to allow for building a solid baseline for molecular barcoding on which ecological and epidemiological studies could be based in future. Moreover, it is already exciting to visualise an idea of using the outputs of NGS, most easily the mt genome sequences, to test their power in addressing the phylogenetic interrelationships of *Diplostomum* spp. when analysed at the amino acid level.

Although the currently utilised *cox1* ‘barcode’ fragment seems useful in assigning individual isolates of *Diplostomum* to both described species and novel molecularly defined lineages, its use for species delineation has a number of disadvantages. First, single molecular markers for robust molecular systematic estimates are insufficient, and multi-gene approaches are preferred; the recently applied coalescent-based approaches [14] and the molecular resources provided here hold significant promise for species delimitation within *Diplostomum*. Secondly, phylogenetic estimates based on the *cox1* ‘barcode’ fragment [8, 14] generally lack nodal support at the internal nodes that define the interrelationships of individual lineages and therefore the phylogenetic utility of this region is limited. Finally, and most importantly, we have shown that the *cox1* gene in fact represents the most conserved protein-coding region of the mt genome of *Diplostomum* spp. (Fig. 1), as already shown for other parasitic flatworms (e.g. [35, 36]) and that the ‘barcode’ region is even more conserved. The comparative mt genome assessment allowed us to identify genes with the greatest interspecific variation that are promising for the development of molecular diagnostic markers for species recognition [35]. Certainly the best candidates are the *nad4* and *nad5* genes that are both relatively long and the least conserved, even when comparing such closely related taxa as *D. spathaceum* and *D. pseudospathaceum*, and thus have the potential to add phylogenetic signal and possibly resolve current taxonomic problems. The use of these new markers in large-scale population studies on *Diplostomum* spp. will offer the advantage of simultaneous species delimitation and assessment of intraspecific genetic variation and thus boost molecular epidemiology studies based on the most accessible life-cycle stages, i.e. the larval forms in the snail and fish populations.

The accelerated development of methods for next-generation biodiversity assessment such as environmental DNA (eDNA) and metabarcoding [37] offer significant promise for large-scale spatial studies related to disease ecology. The first study using water samples to assess the presence of the pathogenic parasite of frogs *Ribeiroia ondatrae* in wetland habitats indicates the high potential for detecting free-living larval stages of macroparasite infectious agents in the aquatic environment [38]. Our study provides a resource for the design of primer pairs targeting very short DNA sequences that would allow the use of NGS tools to identify unambiguously *Diplostomum* spp. in both bulk samples (natural assemblages of metacercariae in fish eyes) and freshwater samples containing DNA of the dispersive free-living larval stages (miracidia, cercariae).

The novel data on mt genomes and nuclear rRNA operons of *Diplostomum* spp. allowed the first genome wide estimation of the phylogenetic relationships of the order Diplostomida, one of the two fundamental lineages from which extant digeneans have diversified [39]. Surprisingly, the currently available mitogenomic data for the Digenea analysed at both the amino acid and nucleotide levels invariably recovered, albeit with much weaker statistical support, the Diplostomidae as a sister lineage of the order Plagiorchiida rather than as a basal lineage of the Diplostomida, the latter inferred by both the benchmark phylogeny of the Digenea of [39] and the current rDNA-based phylogenetic analysis for the set of taxa used in mt genome-based analysis (Fig. 2). This is perhaps the most striking finding given the significance and depth of the basal dichotomy in the Digenea between the Plagiorchiida and Diplostomida on both the molecular [39] and life history levels [40]. If the mt-based phylogeny reflects actual organismal phylogeny, with Diplostomidae as an early diverging lineage within the Plagiorchiida, the seemingly synapomorphic life history characteristics of Diplostomida would need to be reconsidered. For example, the active penetration of a vertebrate host by cercaria in the Diplostomida (with the exception of Brachylaimoidea), or the utilization of tetrapod vertebrates as definitive hosts (with the exception of the Aporocotylidae), would be viewed as plesiomorphies for the Digenea. However, addressing such questions would currently be premature since the mt genomes of *Diplostomum* spp. could be compared solely with those of taxa representing the most derived lineages of both the Diplostomida and the Plagiorchiida. Additional mt genome data, especially for the earlier diverging lineages of the Diplostomida (Brachylaimoidea, Clinostomidae, Aporocotylidae and Spirorchidae) and Plagiorchiida (e.g. Bivesiculoidea, Transversotrematoidea) would allow testing of this hypothesis.

In light of the conflict between nuclear ribosomal and mitochondrial protein estimates of phylogeny, it may be less surprising that mt genome organisation of *Diplostomum* spp. also closely matches that of the nearly perfectly conserved mt genomes of representatives of the Plagiorchiida, rather than that of any representative of the Schistosomatidae, the only members of the order Diplostomida with mt genomes characterised prior to our study. Compared with the mt gene order in all species of the Plagiorchiida, there was a single minor alteration in the mt genomes of *Diplostomum* spp., i.e. a reciprocally switched position of immediately neighbouring genes *trnP* and *trnN* (Fig. 3). This tRNA gene position switch likely represents an autapomorphy for *Diplostomum*, because *trnP* is always found immediately downstream to *trnN* throughout remaining trematodes as well as cestodes, the sister lineage of the trematodes (e.g. [34]). Species of the plagiorchiidan genera *Dicrocoelium*, *Paragonimus* and *Paramphistomum* then display a further common switch of neighbouring *trnE* and *trnG* gene positions; however, this minor reorganisation does not seem to reflect actual cladogenetic events and might have arisen several times during digenean evolution. Contrary to the members of the Plagiorchiida, species of the Schistosomatidae display a significantly altered gene order, including a few tRNA transpositions (as seen in *Trichobilharzia regenti* and two Asian schistosomes) and two larger mt genome region rearrangement events within the more derived African schistosomes (see [17, 41] for details). Further mt genome data for a wider range of taxa, especially for representatives of the remaining families of the Diplostomida (see above), would provide important insights into the patterns and possible mechanisms of mt genome evolution in early divergent digeneans.

## Conclusions

The genus *Diplostomum* represents a taxonomically complex group, with unsatisfactorily resolved species diversity, host-associations and distribution patterns. Application of molecular tools offers a breakthrough to overcome previous problems and obstacles caused by the existence of cryptic diversity and the morphological uniformity of larval stages, especially metacercariae in fish. Our results represent a significant step towards a considerably better understanding of the convoluted systematics of the genus and a valuable resource for marker design that will enhance the development of large-scale biodiversity and molecular epidemiology assessments of these important pathogens in the freshwater environment.

## Additional files

Additional file 1: Figure S1. Generalised life-cycle of *Diplostomum* spp. Additional file 2: Figure S2. Putative secondary structures of the 22 tRNAs identified in the mt genome of *Diplostomum spathaceum*. Additional file 3: Figure S3. Putative secondary structures of the 22 tRNAs identified in the mt genome of *Diplostomum pseudospathaceum*. Additional file 4: Table S1. Codon usage in the mt protein-coding genes of *Diplostomum spathaceum* and *D. pseudospathaceum*.
