# Supplementary figures and images for: Complete mitochondrial genomes and nuclear ribosomal RNA operons of two species of Diplostomum (Platyhelminthes: Trematoda): a molecular resource for taxonomy and molecular epidemiology of important fish pathogens
Source: Parasit Vectors. 2015 Jun 19;8:336. doi: 10.1186/s13071-015-0949-4 (PMC4477422; doi:10.1186/s13071-015-0949-4)

**Additional Figure S1** Generalised life-cycle of *Diplostomum* spp.

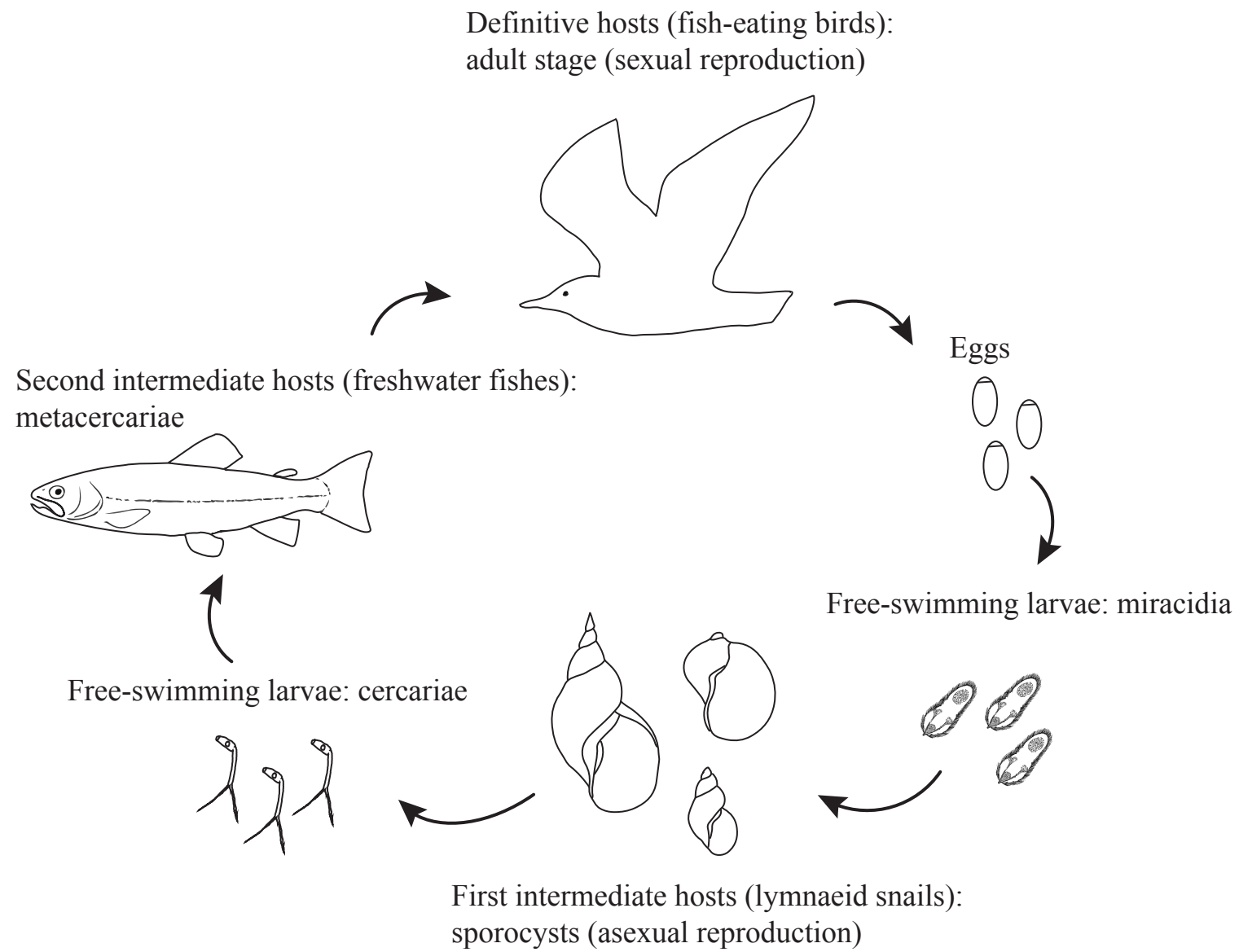

Supplement: Additional file 1: Figure S1. — Generalised life-cycle of Diplostomum spp. [file 13071_2015_949_MOESM1_ESM.pdf]
