# Supplementary material for: Complete mitochondrial genomes and nuclear ribosomal RNA operons of two species of Diplostomum (Platyhelminthes: Trematoda): a molecular resource for taxonomy and molecular epidemiology of important fish pathogens
Source: Parasit Vectors. 2015 Jun 19;8:336. doi: 10.1186/s13071-015-0949-4 (PMC4477422; doi:10.1186/s13071-015-0949-4)

Additional Figure S2 Putative secondary structures of the 22 tRNAs identified in the mt genome of *Diplostomum spathaceum*.

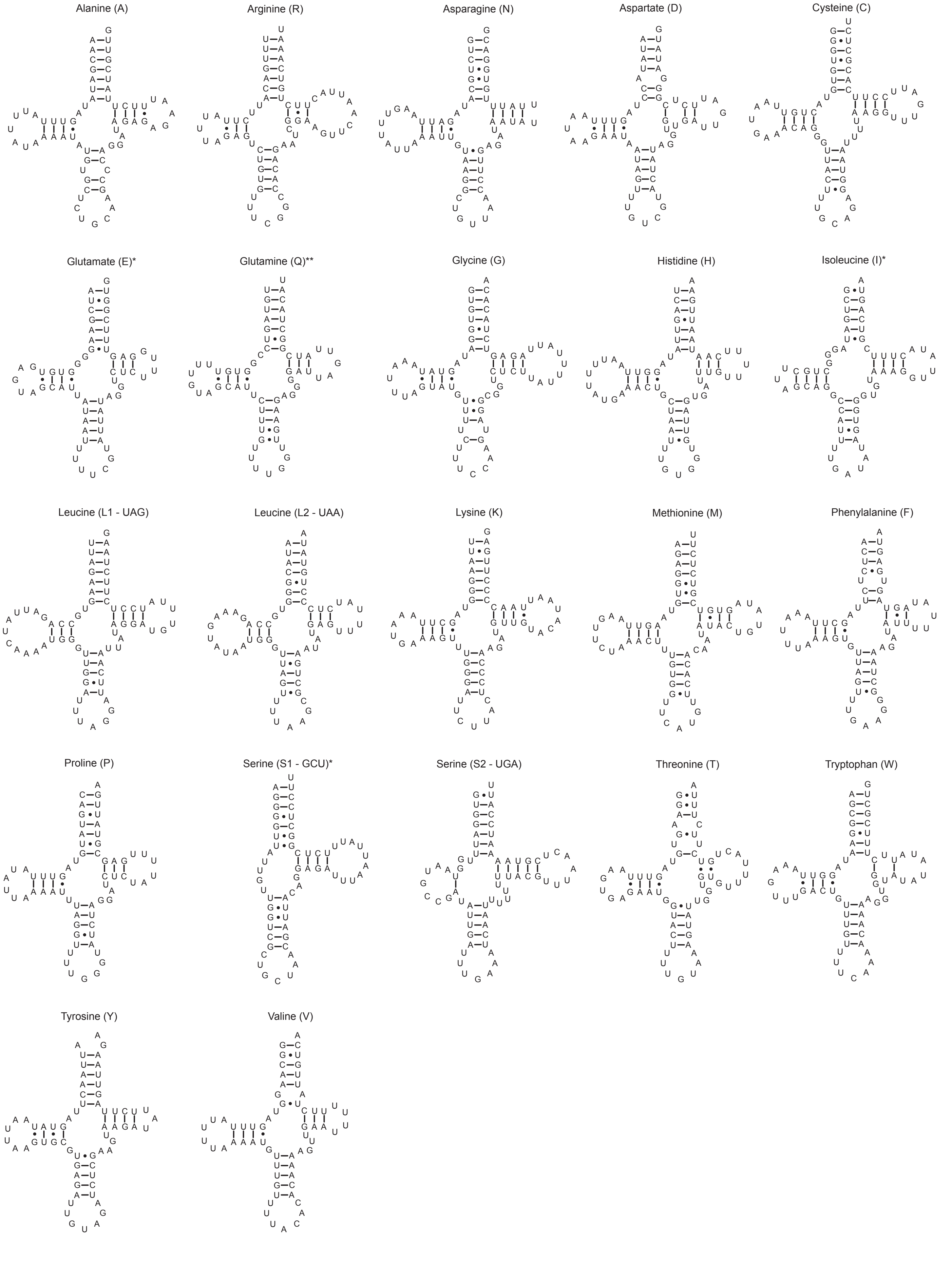

Supplement: Additional file 2: Figure S2. — Putative secondary structures of the 22 tRNAs identified in the mt genome of Diplostomum spathaceum. [file 13071_2015_949_MOESM2_ESM.pdf]
