# Supplementary material for: Complete mitochondrial genomes and nuclear ribosomal RNA operons of two species of Diplostomum (Platyhelminthes: Trematoda): a molecular resource for taxonomy and molecular epidemiology of important fish pathogens
Source: Parasit Vectors. 2015 Jun 19;8:336. doi: 10.1186/s13071-015-0949-4 (PMC4477422; doi:10.1186/s13071-015-0949-4)
Supplement: Additional file 4: Table S1. — Codon usage in the mt protein-coding genes of Diplostomum spathaceum and D. pseudospathaceum. [file 13071_2015_949_MOESM4_ESM.pdf]

Electronic additional file for

Complete mitochondrial genomes and nuclear ribosomal RNA operons of two species of *Diplostomum* (Platyhelminthes: Trematoda): a molecular resource for taxonomy and molecular epidemiology of important fish pathogens

Jan Brabec[1], Aneta Kostadinova[1], Tomáš Scholz[1] and D. Timothy J. Littlewood[2]

[1]Institute of Parasitology, Biology Centre of the Czech Academy of Sciences and Faculty of Science, University of South Bohemia, Branišovská 31, 370 05 České Budějovice, Czech Republic, [2]Department of Life Sciences, Natural History Museum, Cromwell Road, London SW7 5BD, United Kingdom

Additional Table S1 Codon usage in the mt protein-coding genes of *Diplostomum spathaceum* (first line) and *D. pseudospathaceum* (second line) expressed as a total count and percentage (in parentheses) of a given codon in a gene.

Amino acid	Codon	**atp6**	**cob**	**cox1**	**cox2**	**cox3**	**nad1**	**nad2**	**nad3**	**nad4**	**nad4L**	**nad5**	**nad6**
**Non-polar**													
Alanine (A)	GCN	6 (3.5)	15 (4.1)	17 (3.1)	6 (2.9)	4 (1.8)	13 (4.3)	4 (1.3)	0 (0.0)	16 (3.7)	1 (1.1)	20 (3.8)	2 (1.3)
		7 (4.0)	17 (4.6)	18 (3.3)	6 (2.9)	2 (0.9)	10 (3.3)	4 (1.3)	0 (0.0)	13 (3.0)	1 (1.1)	17 (3.2)	1 (0.7)
Isoleucine (I)	ATY	17 (9.8)	24 (6.5)	52 (9.4)	15 (7.3)	16 (7.3)	24 (7.9)	34 (11.4)	8 (6.7)	32 (7.4)	11 (12.5)	42 (7.9)	13 (8.5)
		15 (8.7)	26 (7.0)	53 (9.6)	14 (6.8)	14 (6.4)	28 (9.2)	31 (10.3)	8 (6.7)	27 (6.3)	11 (12.5)	43 (8.1)	15 (9.8)
Leucine (L)	CTN	3 (1.7)	12 (3.2)	17 (3.1)	5 (2.4)	4 (1.8)	8 (2.6)	7 (2.4)	5 (4.2)	14 (3.2)	4 (4.5)	11 (2.1)	5 (3.3)
		2 (1.2)	12 (3.2)	12 (2.2)	5 (2.4)	5 (2.3)	9 (3.0)	7 (2.4)	3 (2.5)	13 (3.0)	3 (3.4)	9 (1.7)	3 (2.0)
Leucine (L)	TTR	30 (17.3)	51 (13.8)	60 (10.8)	11 (5.4)	28 (12.8)	39 (12.9)	33 (11.1)	13 (10.9)	64 (14.8)	19 (21.6)	77 (14.6)	15 (9.8)
		31 (17.9)	49 (13.2)	66 (11.9)	11 (5.4)	25 (11.5)	38 (12.5)	35 (11.7)	15 (12.6)	68 (15.7)	22 (25.0)	79 (14.9)	17 (11.1)
Methionine (M)	ATR	4 (2.3)	8 (2.2)	25 (4.5)	8 (3.9)	7 (3.2)	10 (3.3)	6 (2.0)	1 (0.8)	8 (1.8)	2 (2.3)	12 (2.3)	2 (1.3)
		4 (2.3)	8 (2.2)	25 (4.5)	7 (3.4)	6 (2.8)	9 (3.0)	7 (2.3)	2 (1.7)	6 (1.4)	2 (2.3)	12 (2.3)	2 (1.3)
Phenylalanine (F)	TTY	28 (16.2)	32 (8.6)	55 (9.9)	15 (7.3)	29 (13.3)	37 (12.2)	52 (17.5)	20 (16.8)	57 (13.2)	8 (9.1)	78 (14.7)	25 (16.3)
		25 (14.5)	33 (8.9)	55 (9.9)	14 (6.8)	31 (14.2)	39 (12.9)	57 (19.0)	18 (15.1)	58 (13.4)	7 (8.0)	74 (14.0)	23 (15.0)
Proline (P)	CCN	7 (4.0)	11 (3.0)	26 (4.7)	7 (3.4)	6 (2.8)	5 (1.7)	5 (1.7)	3 (2.5)	11 (2.5)	0 (0.0)	5 (0.9)	3 (2.0)
		7 (4.0)	11 (3.0)	26 (4.7)	7 (3.4)	6 (2.8)	5 (1.7)	5 (1.7)	2 (1.7)	10 (2.3)	0 (0.0)	5 (0.9)	2 (1.3)
Tryptophan (W)	TGR	2 (1.2)	10 (2.7)	18 (3.3)	5 (2.4)	7 (3.2)	12 (4.0)	12 (4.0)	3 (2.5)	14 (3.2)	1 (1.1)	11 (2.1)	5 (3.3)
		2 (1.2)	10 (2.7)	18 (3.3)	5 (2.4)	7 (3.2)	11 (3.6)	12 (4.0)	3 (2.5)	14 (3.2)	1 (1.1)	12 (2.3)	5 (3.3)
Valine (V)	GTN	16 (9.2)	43 (11.6)	40 (7.2)	23 (11.2)	25 (11.5)	30 (9.9)	25 (8.4)	15 (12.6)	39 (9.0)	9 (10.2)	57 (10.8)	17 (11.1)
		20 (11.6)	42 (11.4)	38 (6.9)	25 (12.2)	27 (12.4)	27 (8.9)	21 (7.0)	15 (12.6)	40 (9.3)	9 (10.2)	56 (10.6)	17 (11.1)

**Polar**													
Asparagine (N)	AAY	5 (2.9)	9 (2.4)	16 (2.9)	6 (2.9)	4 (1.8)	8 (2.6)	6 (2.0)	6 (5.0)	10 (2.3)	3 (3.4)	12 (2.3)	4 (2.6)
		3 (1.7)	10 (2.7)	17 (3.1)	4 (2.0)	5 (2.3)	7 (2.3)	5 (1.7)	7 (5.9)	12 (2.8)	3 (3.4)	12 (2.3)	3 (2.0)
Cysteine (C)	TGY	3 (1.7)	7 (1.9)	8 (1.4)	7 (3.4)	6 (2.8)	12 (4.0)	12 (4.0)	2 (1.7)	18 (4.2)	2 (2.3)	19 (3.6)	6 (3.9)
		4 (2.3)	7 (1.9)	8 (1.4)	7 (3.4)	6 (2.8)	12 (4.0)	12 (4.0)	2 (1.7)	19 (4.4)	2 (2.3)	20 (3.8)	6 (3.9)
Glutamine (Q)	CAR	0 (0.0)	4 (1.1)	1 (0.2)	3 (1.5)	1 (0.5)	4 (1.3)	1 (0.3)	1 (0.8)	1 (0.2)	1 (1.1)	3 (0.6)	0 (0.0)
		0 (0.0)	3 (0.8)	1 (0.2)	3 (1.5)	1 (0.5)	4 (1.3)	1 (0.3)	1 (0.8)	1 (0.2)	1 (1.1)	3 (0.6)	0 (0.0)
Glycine (G)	GGN	11 (6.4)	22 (5.9)	47 (8.5)	14 (6.8)	17 (7.8)	20 (6.6)	14 (4.7)	5 (4.2)	29 (6.7)	4 (4.5)	36 (6.8)	11 (7.2)
		11 (6.4)	20 (5.4)	47 (8.5)	15 (7.3)	16 (7.3)	20 (6.6)	17 (5.7)	6 (5.0)	26 (6.0)	4 (4.5)	36 (6.8)	11 (7.2)
Serine (S)	AGN	2 (1.2)	22 (5.9)	22 (4.0)	6 (2.9)	10 (4.6)	11 (3.6)	19 (6.4)	5 (4.2)	21 (4.9)	5 (5.7)	18 (3.4)	10 (6.5)
		3 (1.7)	22 (5.9)	20 (3.6)	8 (3.9)	10 (4.6)	12 (4.0)	19 (6.4)	2 (1.7)	25 (5.8)	5 (5.7)	21 (4.0)	11 (7.2)
Serine (S)	TCN	11 (6.4)	25 (6.8)	36 (6.5)	8 (3.9)	12 (5.5)	15 (5.0)	29 (9.8)	7 (5.9)	31 (7.2)	3 (3.4)	44 (8.3)	12 (7.8)
		10 (5.8)	25 (6.8)	35 (6.3)	9 (4.4)	12 (5.5)	15 (5.0)	31 (10.3)	9 (7.6)	32 (7.4)	3 (3.4)	48 (9.1)	13 (8.5)
Threonine (T)	ACN	6 (3.5)	13 (3.5)	23 (4.2)	10 (4.9)	5 (2.3)	8 (2.6)	8 (2.7)	3 (2.5)	9 (2.1)	6 (6.8)	16 (3.0)	2 (1.3)
		7 (4.0)	12 (3.2)	23 (4.2)	10 (4.9)	5 (2.3)	9 (3.0)	7 (2.3)	2 (1.7)	12 (2.8)	5 (5.7)	13 (2.5)	2 (1.3)
Tyrosine (Y)	TAY	5 (2.9)	25 (6.8)	29 (5.2)	14 (6.8)	16 (7.3)	18 (5.9)	17 (5.7)	10 (8.4)	24 (5.5)	0 (0.0)	27 (5.1)	12 (7.8)
		5 (2.9)	26 (7.0)	29 (5.2)	14 (6.8)	17 (7.8)	18 (5.9)	16 (5.3)	11 (9.2)	23 (5.3)	0 (0.0)	31 (5.9)	13 (8.5)
**Acidic**													
Aspartate (D)	GAY	2 (1.2)	7 (1.9)	18 (3.3)	14 (6.8)	4 (1.8)	4 (1.3)	2 (0.7)	2 (1.7)	10 (2.3)	1 (1.1)	11 (2.1)	1 (0.7)
		2 (1.2)	7 (1.9)	17 (3.1)	13 (6.3)	4 (1.8)	4 (1.3)	3 (1.0)	2 (1.7)	8 (1.9)	1 (1.1)	9 (1.7)	1 (0.7)
Glutamate (E)	GAR	3 (1.7)	7 (1.9)	5 (0.9)	7 (3.4)	7 (3.2)	8 (2.6)	3 (1.0)	5 (4.2)	5 (1.2)	4 (4.5)	8 (1.5)	2 (1.3)
		3 (1.7)	7 (1.9)	5 (0.9)	8 (3.9)	9 (4.1)	8 (2.6)	3 (1.0)	6 (5.0)	6 (1.4)	4 (4.5)	7 (1.3)	2 (1.3)
**Basic**													
Arginine (R)	CGN	8 (4.6)	7 (1.9)	11 (2.0)	5 (2.4)	1 (0.5)	8 (2.6)	1 (0.3)	1 (0.8)	8 (1.8)	2 (2.3)	9 (1.7)	2 (1.3)
		8 (4.6)	7 (1.9)	11 (2.0)	5 (2.4)	1 (0.5)	8 (2.6)	1 (0.3)	1 (0.8)	7 (1.6)	2 (2.3)	10 (1.9)	2 (1.3)
Histidine (H)	CAY	3 (1.7)	8 (2.2)	16 (2.9)	6 (2.9)	7 (3.2)	1 (0.3)	2 (0.7)	1 (0.8)	5 (1.2)	1 (1.1)	6 (1.1)	2 (1.3)
		3 (1.7)	8 (2.2)	16 (2.9)	6 (2.9)	7 (3.2)	2 (0.7)	1 (0.3)	1 (0.8)	5 (1.2)	1 (1.1)	5 (0.9)	2 (1.3)
Lysine (K)	AAR	0 (0.0)	8 (2.2)	10 (1.8)	9 (4.4)	2 (0.9)	8 (2.6)	5 (1.7)	2 (1.7)	6 (1.4)	0 (0.0)	6 (1.1)	1 (0.7)
		0 (0.0)	8 (2.2)	12 (2.2)	8 (3.9)	2 (0.9)	8 (2.6)	5 (1.7)	2 (1.7)	6 (1.4)	0 (0.0)	6 (1.1)	1 (0.7)

International Union of Pure and Applied Chemistry (IUPAC) codes (N=A, G, C or T; Y=C or T; R=A or G) were used.
